# Time-Transgressive Nature of the Magnetic Susceptibility Record across the Chinese Loess Plateau at the Pleistocene/Holocene Transition

**DOI:** 10.1371/journal.pone.0133541

**Published:** 2015-07-17

**Authors:** Yajie Dong, Naiqin Wu, Fengjiang Li, Linpei Huang, Wenwen Wen

**Affiliations:** 1 Key Laboratory of Cenozoic Geology and Environment, Institute of Geology and Geophysics, Chinese Academy of Sciences, Beijing, China; 2 University of the Chinese Academy of Sciences, Beijing, China; 3 Key Laboratory of Plateau Lake Ecology and Global Change, College of Tourism and Geography, Yunnan Normal University, Kunming, China; Institute of Botany, CHINA

## Abstract

The loess stratigraphic boundary at the Pleistocene/Holocene transition defined by the magnetic susceptibility (MS) has previously been assumed to be synchronous with the Marine Isotope Stage (MIS) 2/1 boundary, and approximately time-synchronous at different sections across the Chinese Loess Plateau (CLP). However, although this assumption has been used as a basis for proxy-age model of Chinese loess deposits, it has rarely been tested by using absolute dating methods. In this study, we applied a single-aliquot regenerative-dose (SAR) protocol to the 45–63 μm quartz grain-size fraction to derive luminescence ages for the last glacial and Holocene sections of three loess sections on a transect from southeast to northwest across the CLP. Based on the 33 closely spaced optically stimulated luminescence (OSL) samples from the three sections, OSL chronologies were established using a polynomial curve fit at each section. Based on the OSL chronology, the timing of the Pleistocene/Holocene boundary, as defined by rapid changes in MS values, is dated at ~10.5 ka, 8.5 ka and 7.5 ka in the Yaoxian section, Jingchuan and Huanxian sections respectively. These results are clearly inconsistent with the MIS 2/1 boundary age of 12.05 ka, and therefore we conclude that the automatic correlation of the Pleistocene/Holocene transition, as inferred from the MS record, with the MIS 2/1 boundary is incorrect. The results clearly demonstrate that the marked changes in MS along the southeast to northwest transect are time-transgressive among the different sites, with the timing of significant paleosol development as indicated by the MS record being delayed by 3–4 ka in the northwest compared to the southeast. Our results suggest that this asynchronous paleosol development during the last deglacial was caused by the delayed arrival of the summer monsoon in the northwest CLP compared to the southeast.

## Introduction

The loess-paleosol sequences of the Chinese Loess Plateau (CLP) provide a continuous, high-resolution record of climate change throughout the Quaternary period, unparalleled in other terrestrial sediment archives [1–14]. Proxy climate data recorded in loess sequences, such as the magnetic susceptibility and the sediment grain size distribution, correlate well with the orbitally-tuned marine oxygen isotope record and exhibit clear glacial and interglacial cycles [1,5]. Thus various attempts have been made to use these correlations to generate proxy age models to provide a continental timescale for the Quaternary [3,14]. These age models are generally accepted and provide reliable long-term timescale [9,12,15].

There are two important assumptions made with regard to the stratigraphic boundaries, or age control points, used in developing such proxy age models for Chinese loess deposits. (i) The major changes in a given proxy record (e.g. magnetic susceptibility, MS) are assumed to be time-synchronous with those in the marine oxygen isotope (MIS) record [14]. For example, the horizon of rapid change in MS at the Pleistocene/Holocene transition is usually regarded as being time-equivalent to the MIS2/1 boundary (12.05 ka). Thus the MIS boundaries are applied to derive a loess chronology using a ‘wiggle-matching’ approach [14]. (ii) The second assumption is that changes in the loess proxy records are isochronous in potentially widely separated sections on the CLP [5]. If this assumption is correct then sites can be correlated and chronologies established across the entire CLP. Although there are significant variations in sediment accumulation rate, and thus in the thickness of a given stratigraphic unit, across the CLP, proxy records such as MS and grain size are quite similar [1,3,8,16]. The foregoing assumptions are widely accepted and have been applied to develop chronologies of the loess-paleosol sequences on a long timescale [9,12,15]. However on a finer time scale, recent investigations have highlighted age discrepancies based on different dating method between these age models and those derived from absolute dating techniques [17–23], indicating that these assumptions may not always be correct.

In particular, the first assumption has been challenged by recent studies based on absolute age dating. Lai and Wintle (2006) pointed out that the Pleistocene/Holocene boundary based on the MS record is dated to ~9.8 ka in the Yuanbao loess section, located in the west of the CLP, which is clearly asynchronous with the MIS2/1 boundary (12.05 ka) [18]. Similarly, Lu et al. (2006) found that the MS-defined boundary at the Huanxian and Xifeng sections in the north-central CLP is ~7–9 ka, significantly younger than MIS2/1 [24]. The sites which have been dated using absolute dating techniques are mainly located in the north-central and western CLP. With regard to the second assumption, paleoclimatologists cannot be certain that the age-control points are reliable between different sites, because few sites have been investigated to compare high resolution changes in MS in the south and central parts of the CLP. It is noteworthy that the second assumption is similar to that involved in the use of the marine oxygen isotope record for stratigraphic correlation, i.e. that the mixing time of the oceans is sufficiently short (~10^3^yr) to result in essentially synchronous oxygen isotopic variations in deep-sea cores which may be separated by thousands of kilometers [25–27]. However, recent research suggests that changes in monsoon intensity have been asynchronous across China causing the terrestrial proxy record in different region to lag or lead ice volume and marine δ^18^O changes [28,29]. This implies that the terrestrial response to climate change documented in proxy data is not much more rapid than has been assumed. Although the concept of the asynchronous nature of changes in monsoon intensity across China remains controversial [30], an increasing number of researchers have questioned the synchroneity of the loess/paleosol boundaries across the CLP. However, although there is increasing evidence that this second assumption may be incorrect, it has not been investigated systematically using reliable absolute dating at multiple sites across the CLP.

Given the growing uncertainty in the reliability of proxy-based age models for Chinese loess, it has been suggested that independent direct dating is the only means by which an effective chronology of deposition and climate history may be gained. Optically stimulated luminescence (OSL) dating techniques are particularly suitable for application to Chinese loess due to its high content of eolian sedimentary feldspar or quartz grains [31,32]. One of the advantages of OSL dating is that the ages obtained represent the last sunlight exposure event before deposition; thus, high resolution OSL dating may shed light on the dynamics of dust accumulation and help test the assumptions listed above. Recently, OSL dating has been applied to eolian dust deposited during the last glacial/interglacial cycle [17–20,23,24,32–40]. However, relatively few studies have utilized independent dates obtained at the resolution required systematically to address the issue of boundary ages and the synchroneity of stratigraphic boundaries.

The present paper reports the result of OSL dating of three late Quaternary loess sequences along a northwest to southeast transect covering the main part of the CLP. The main aims were (i) to use OSL dating to establish an accurate and precise chronology for these loess sequences, thus enabling the assessment of the age of the Pleistocene/Holocene boundary, as defined by rapid change in MS; and (ii) to examine whether the boundary ages defined by the MS record are synchronous with the MIS record among several sites across the CLP, and thereby to evaluate the assumptions implicit in the proxy-based age modelling approach.

## Materials and Methods

### Study area and sampling

**Fig 1:**
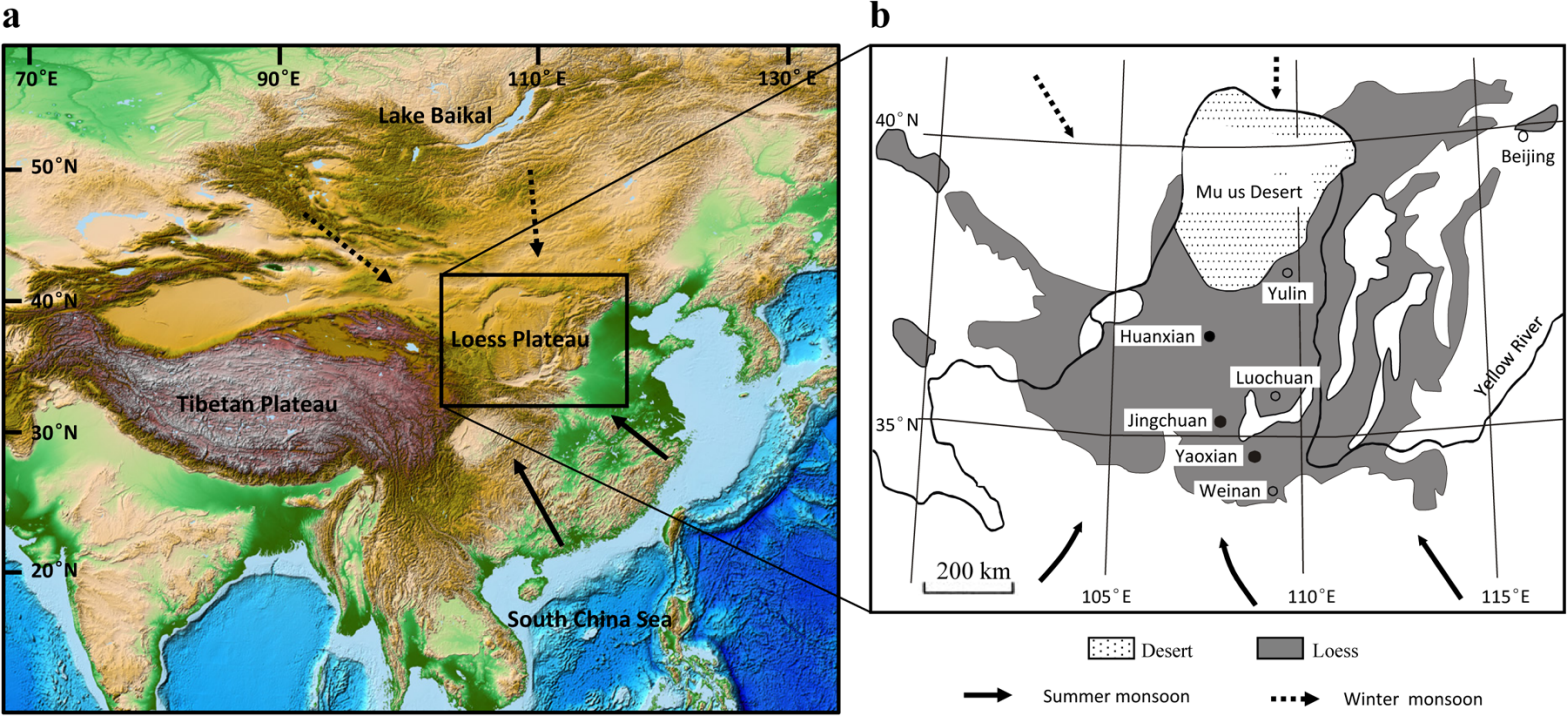
(a) Location of the Chinese Loess Plateau (CLP) in High Asia (map modified from NOAA; http://www.ngdc.noaa.gov/mgg/global/global.html). **(b) The central Chinese Loess Plateau and location of the sites referenced in the text** (modified from Wu et al., 2002 [7]). The solid arrows indicate the pathways of the East Asian summer monsoons; dashed arrows indicate the pathways of the East Asian winter monsoons.

The three loess sections (Huanxian, Jingchuan, and Yaoxian) are located along a NW-SE transect across the Chinese Loess Plateau (CLP) (Fig 1), which exhibits the steepest climatic gradient in the region. The CLP is located in the East Asian monsoon region, and in summer, the summer monsoon carries warm, moist air masses to the CLP, causing heavy rainfall and strong soil development. Accordingly, most of the annual rainfall (over 50%) in the area is concentrated from July to September. In winter, winter monsoon winds from the Siberian High prevail over the region, resulting in a cold and dry climate [41]. Presently, the mean annual precipitation (MAP) of the three study sites ranges from ~350 mm to ~570 mm [41]. The studied loess sequences include the S0 and L1 units which were deposited since MIS 2.

**Fig 2:**
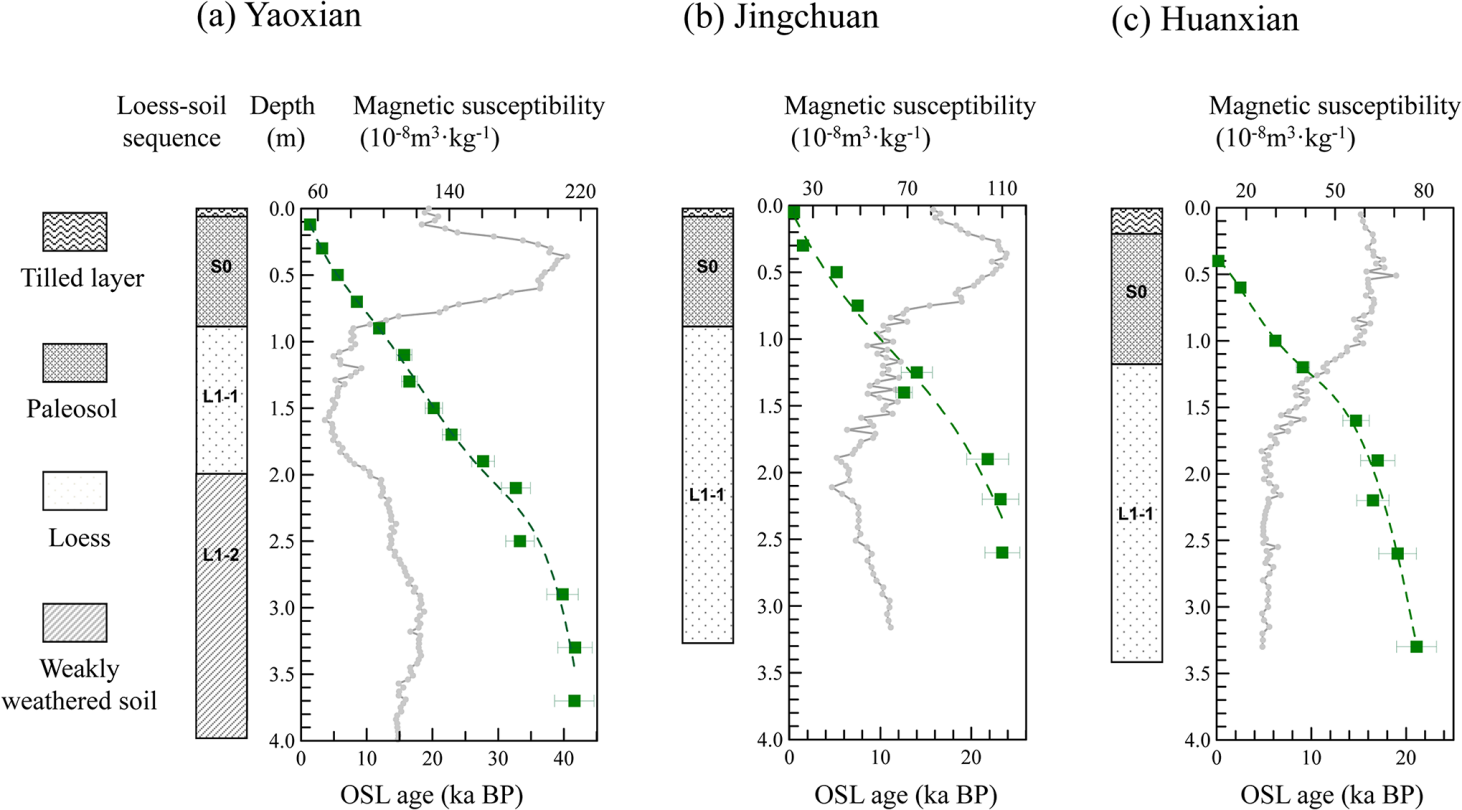
Pedostraigraphy, magnetic susceptibility and OSL ages versus depth for the loess sections at Yaoxian (a), Jingchuan (b) and Huanxian (c) since the last glacial.

The Yaoxian (YX) section (34°53′N, 108°58′E) is located near the northern edge of Guanzhong Basin, about 100 km north of Xi’an. The area has a mean annual temperature (MAT) of ~12.3°C and a mean annual precipitation (MAP) of ~580 mm. The sampled part of the section consists of the S0 paleosol unit, ~0.9 m thick, and the upper 3.3m part of the L1 loess unit which is divided into sub-units L1-1 and L1-2 (Fig 2A). The Jingchuan (JC) section (35°15′N, 107°43′E) is located ~100 km north of Yaoxian near the city of Changwu, Shaanxi Province. The area has a MAT of ~9.5°C and a MAP of ~550 mm. The 3.2 m long record comprises an undisturbed S0 paleosol of about 0.8 m thickness and the upper 2.4 m of the L1 loess unit (Fig 2B). The northernmost section, Huanxian (HX) (36°32′N, 107°20′E), is located in the northernmost part of the Loess Plateau near the city of Huanxian, Gansu Province. The MAT of the area is ~8.3°C and MAP of ~350 mm. The investigated 3.3 m long sequence comprises the S0 paleosol unit, ~1.2 m thick, and the upper ~2.1 m of the L1 loess unit (Fig 2C). The uppermost 30 cm of this section was not sampled because of apparent agricultural disturbance. No necessary permits for the described field investigations were needed.

In the field, a total of 328 samples for magnetic susceptibility measurements were first collected from the 3 sections at a 3 cm interval. Then, a total of 33 samples (HX = 9, CW = 9, YX = 15) for OSL dating were collected. The OSL samples were taken by hammering 20-cm-long stainless steel tubes (5 cm diameter) into the freshly cleaned surface. The OSL sampling intervals were 20 cm for the S0 paleosol unit and 20–40 cm for the upper part of the L1 loess unit.

### Magnetic susceptibility measurement and data analysis

The magnetic susceptibility (MS) of the three sections was measured in the laboratory on air-dried samples using a Bartington Instruments MS2 susceptibility meter following the procedures of Liu (1985) [1] and Kukla et al. (1988) [42]. Next the MS data were plotted and analyzed visually to determine the boundary between the S0 paleosol unit and the L1 loess unit at the Pleistocene/Holocene transition. Although it is difficult to locate the boundary completely objectively, in our study we used the point of maximum rate-of-change in the MS record–as proposed by Porter and An (1995) [14]. The MIS boundary referred to as a termination is also defined as the most rapid rate of change of the δ^18^O record [43], and is used widely by many researchers [18,24]. To determine the position as accurately as possible, we applied the rate-of-change analysis method [44–47], which is quantified by determining the total amount of change in MS per unit of time between adjacent samples. Finally, the age of the boundary as defined by MS was dated and comparisons made between the sites.

### OSL measurements

Thirty three samples were collected to establish the chronology of loess deposition for the three sections using optical stimulated luminescence dating (OSL). The sample tubes were processed under subdued red light conditions in the laboratory. The sediments at the two ends of the tube, which may have been exposed to daylight during sampling, were retained for radioisotope (U, Th and K) concentration analysis and for water content measurement. The remainder of the unexposed loess sample was prepared for quartz OSL equivalent dose (De) determination.

Subsequently the 45–63μm grain size fraction was extracted using laboratory pretreatment procedures for loess as described in Qin and Zhou (2007) [48]. The samples (approximately 50 g) were first treated with 30% H_2_O_2_ and 30% HCl to remove organic materials and carbonates, respectively. This fraction was then etched using fluosicilic acid for about two weeks to dissolve feldspars. The quartz extracts were then checked for purity. The feldspar contamination has been efficiently removed and the samples were considered pure enough as indicated by the characteristic peak at 110 of TL glow curve of quartz and low (<3%) ratio of initial IRSL to blue OSL signals [49]. If a sample failed the purity test, the etching by fluosicilic acid was repeated. Quartz grains were then fixed onto an area of 0.6 cm diameter on 1.0 cm diameter stainless steel discs using silicone oil, and used for equivalent dose (D_e_) determination using the SAR procedure [50] and through construction of standard growth curves [51] using a Riso TL/OSL Reader model 15. The measurements were made at the luminescence dating laboratory at Peking University, China.

Dose rates were calculated using U, Th, and K contents. The concentrations of U and Th were measured using neutron activation analysis (NAA). The modern water content was expressed as percentage of the dry weight and was adopted for the dose rate calculation. The loess chronology was established by performing a polynomial fit through a number of successive OSL dates.

## Results

### Optical stimulated luminescence results and sediment chronology

**Fig 3:**
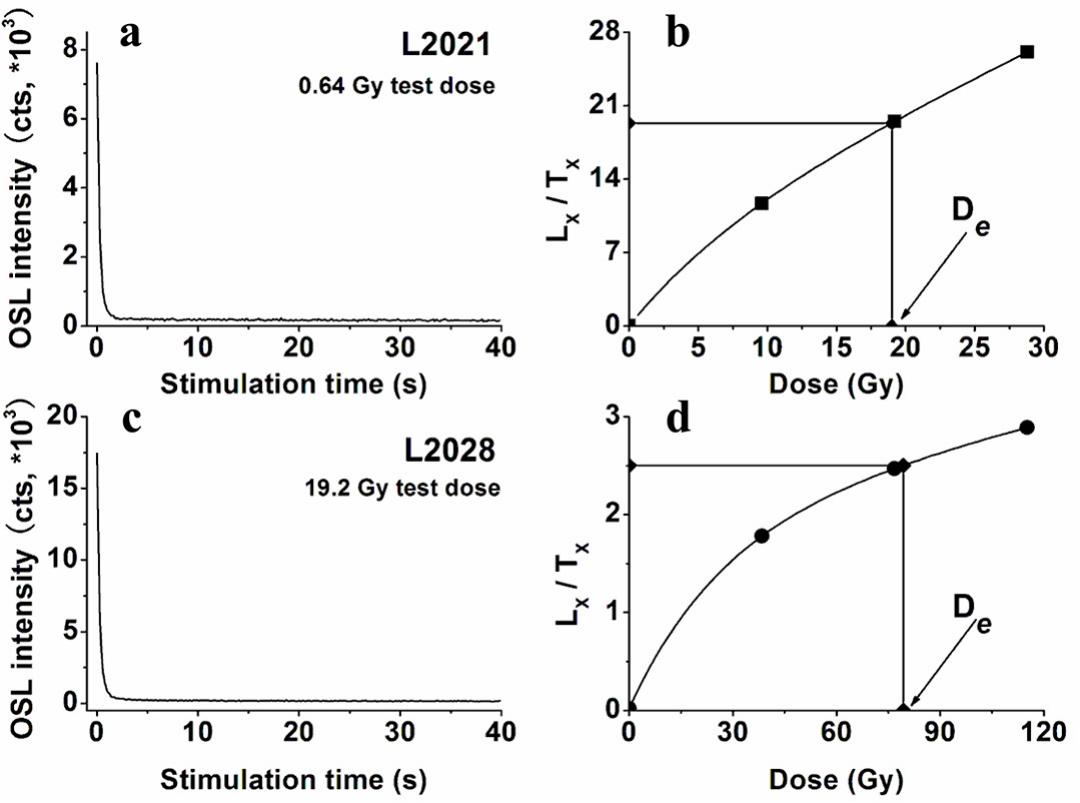
Equivalent dose (De) determination for two samples, L2021 (a, b) and L2028 (c, d), using the SAR protocol. (a) and (c), decay curves of natural OSL intensity. (b) and (d), representative SAR growth curve for a single aliquot of fine-grained quartz.

The typical OSL decay curve can be decomposed into three components: the OSL signal is dominated by fast component, of which the fast component contributes about 95% in the first 0.64s of the 40s stimulation (Fig 3). The regenerative growth curves were modeled by using the exponential plus linear form. For most of the aliquots the regenerative growth curves demonstrate that (1) the recuperation is close to zero; (2) the recycling ratio is within 10% of unity; (3) the signal is not saturated at the level of the natural signal. Test doses of 19.2Gy and 0.64 Gy were used for samples L2028 and L2021, respectively (Fig 3). Recuperation is lower than 3% for all samples, which indicates insignificant charge transfer during the equivalent dose determination. These favorable luminescence characteristics for a sufficient number of aliquots prove that reliable equivalent dose values for loess can be determined using the SAR protocol. A successful dose recovery test indicates the usefulness of the experimental condition of 240°C preheat combined with a cut-heat of 220°C. This preheat strategy was applied to samples from the Yaoxian section. Likewise, experimental conditions of 240°C preheat combined with a cut-heat of 220°C were used for the Jingchuan and Huanxian samples. Table 1 summarizes all of the dose rate, equivalent doses and water content data, and the derived optical ages. These testing results of OSL dating show robust performances, and the chronologies of three sections were then established by using a 5-degree polynomial curve fit.

**Table 1:** Data related to OSL age determinations of 33 samples from the Yaoxian, Jingchuan and Huanxian sections.

Lab ID	Field ID	Depth (m)	Stratigraphy	K (%)	Uranium (ppm)	Thorium (ppm)	Water content (%)	Dose rate (Gy/ka)	De (Gy)	Age (ka)
L2019	10YXB-0.12	0.12	S0	2.00±0.06	2.31±0.10	11.9±0.33	15±5	3.38±0.2	4.66±0.32	1.38±0.12
L2020	10YXB-0.30	0.3	S0	2.08±0.06	2.5±0.11	13.1±0.35	15±5	3.55±0.21	11.39±0.44	3.21±0.23
L2021	10YXB-0.50	0.5	S0	2.11±0.06	2.37±0.10	13.2±0.36	15±5	3.53±0.21	19.65±0.38	5.56±0.35
L2022	10YXB-0.70	0.7	S0	2.23±0.06	2.59±0.11	14.1±0.38	15±5	3.75±0.23	31.85±0.86	8.5±0.58
L2023	10YXB-0.90	0.9	S0	1.81±0.06	2.38±0.10	10.7±0.30	15±5	3.09±0.19	36.64±1.60	11.9±0.89
L2024	10YXB-1.10	1.1	L1-1	1.83±0.06	2.18±0.10	10.2±0.29	20±5	2.87±0.17	45.13±2.21	15.7±1.2
L2025	10YXB-1.30	1.3	L1-1	1.99±0.06	2.36±0.10	11.3±0.32	20±5	3.12±0.18	51.48±2.35	16.5±1.2
L2026	10YXB-1.50	1.5	L1-1	1.81±0.06	2.31±0.10	10.0±0.29	20±5	2.86±0.17	57.85±1.40	20.2±1.3
L2027	10YXB-1.70	1.7	L1-1	1.82±0.06	2.16±0.10	10.3±0.29	20±5	2.85±0.17	65.22±0.99	22.9±1.4
L2028	10YXB-1.90	1.9	L1-1	1.86±0.06	2.22±0.10	10.5±0.29	20±5	2.91±0.17	80.49±1.95	27.7±1.74
L2029	10YXB-2.10	2.1	L1-1	1.99±0.06	2.45±0.11	11.9±0.33	25±5	3.02±0.17	98.67±3.46	32.7±2.19
L2030	10YXB-2.50	2.5	L1-1	1.99±0.06	2.4±0.10	11.4±0.32	25±5	2.97±0.17	98.75±3.16	33.3±2.16
L2031	10YXB-2.90	2.9	L1-2	1.94±0.06	2.32±0.10	11.3±0.32	25±5	2.9±0.17	115.35±2.19	39.8±2.39
L2032	10YXB-3.30	3.3	L1-2	2.00±0.06	2.24±0.10	11.7±0.33	25±5	2.94±0.17	122.73±3.49	41.7±2.64
L2033	10YXB-3.70	3.7	L1-3	1.98±0.06	2.48±0.11	11.9±0.33	25±5	2.99±0.17	123.96±5.34	41.6±3.0
L2056	11HX 0.4	0.4	S0	1.83±0.06	2.6±0.1	11.7±0.33	15±5	3.24±0.2	0.62±0.12	0.19±0.04
L2050	11HX 0.6	0.6	S0	1.84±0.06	2.48±0.1	11.4±0.32	15±5	3.19±0.19	8.13±0.32	2.5±0.2
L2051	11HX 1.0	1.0	S0	1.82±0.06	2.5±0.1	10.4±0.29	15±5	3.10±0.18	19.2±0.8	6.2±0.4
L2052	11HX 1.2	1.2	S0	1.81±0.06	2.31±0.09	10.2±0.29	15±5	3.03±0.18	27.6±1.4	9.1±0.7
L2053	11HX 1.6	1.6	L1	1.85±0.06	2.43±0.09	9.45±0.26	15±5	3.03±0.18	44.6±3.4	14.7±1.4
L2057	11HX 1.9	1.9	L1	1.9±0.06	2.26±0.09	10.7±0.30	20±5	2.96±0.17	50.5±4.3	17.0±1.8
L2058	11HX 2.2	2.2	L1	1.81±0.06	2.72±0.11	11.2±0.31	25±5	2.89±0.17	47.6±4.1	16.5±1.7
L2054	11HX 2.6	2.6	L1	1.9±0.06	2.3±0.09	10.3±0.29	25±5	2.80±0.16	53.5±4.8	19.1±2.0
L2055	11HX 3.3	3.3	L1	1.86±0.06	3.07±0.12	12.2±0.34	25±5	3.05±0.18	64.3±5.3	21.1±2.1
L2059	11CW 0.05	0.05	S0	2.05±0.07	2.46±0.1	12±0.34	15±5	3.50±0.20	1.76±0.1	0.50±0.04
L2060	11CW 0.3	0.3	S0	2.18±0.07	2.46±0.1	12.7±0.36	15±5	3.59±0.22	5.26±0.24	1.5±0.1
L2066	11CW 0. 5	0.5	S0	2.17±0.07	2.7±0.11	13±0.36	15±5	3.65±0.22	18.77±0.8	5.2±0.4
L2061	11CW 0.75	0.75	S0	2.03±0.06	2.47±0.1	12±0.34	15±5	3.39±0.20	25.5±1.2	7.5±0.6
L2062	11CW 1.25	1.25	L1	1.81±0.06	2.21±0.09	10.9±0.31	15±5	3.05±0.18	42.8±4.5	14.0±1.7
L2063	11CW 1.4	1.4	L1	1.9±0.06	2.38±0.09	11.5±0.32	20±5	3.06±0.18	38.6±1.6	12.6±0.9
L2064	11CW 1.9	1.9	L1	1.89±0.06	2.53±0.1	11.1±0.31	20±5	3.05±0.18	66.5±5.8	21.8±2.3
L2067	11CW 2.2	2.2	L1	1.86±0.06	2.19±0.09	11.4±0.32	25±5	2.83±0.16	65.6±4.3	23.2±2.0
L2068	11CW 2.6	2.6	L1	1.87±0.06	2.31±0.09	11±0.31	25±5	2.83±0.16	66.3±4.1	23.4±1.9

Fig 2 illustrates the pedostratigraphy, and the results of MS and OSL measurements for the three sections. The 15 OSL ages obtained at a 20 cm interval at the Yaoxian section demonstrate that there was a continuous record of dust deposition from 42 ka to 1.3 ka, corresponding to a depth interval of 4.0 m. Both the equivalent dose and OSL ages increase monotonically down section. From 370–190 cm, ~42 to ~32 ka, an interval of relatively high sediment accumulation rate is recorded which corresponds to L1-2 (Fig 2A). The uppermost 190 cm of the section, the last 29 ka, exhibits a significantly lower and relatively constant sediment accumulation rate.

The dated record of loess sedimentation at Jingchuan extends from 24 to 0.5 ka (Fig 2B). Below 50 cm, two distinct sediment accumulation rate intervals can be distinguished. A prolonged rapid phase occurs from 22 to 10 ka, and a phase of significantly lower rates occurred between 9 and 1 ka.

The Huanxian section provides a 22 ka record of loess deposition (Fig 2C). At least two major phases of sediment loess accumulation can be distinguished. The interval from ~22–15 ka, corresponding to the depth interval of 350–160 cm, exhibits a sediment accumulation rate of ~25 cm/ka. However, the uppermost 160 cm of loess was deposited at a much lower rate of ~10 cm/ka.

In summary, the high-resolution OSL-dated loess records of the three sections enable the location of the Pleistocene/Holocene boundary to be determined, as defined by the magnetic susceptibility records.

### Locating the Pleistocene/Holocene boundary

The Pleistocene/Holocene transition is clearly expressed in the magnetic susceptibility (MS) records of the three sections. The S0 paleosol is characterized by dramatically higher MS values than the underlying upper part of loess unit L1. To obtain a precise determination of the boundary at the P/H transition, we used the rate-of-change analysis described in method section. This analysis estimates the total amount of change in MS per unit of time between adjacent samples [44–47]. We performed the rate-of-change analysis on the three profiles to locate the boundary. At Yaoxian, the MS values increased sharply from 50–100×10^-8^m^3^/kg to 150–220×10^-8^m^3^/kg after 12 ka, coincident with a colour change of the loess which was observed in the field. Based on the rate-of-change analysis, the most rapid change in MS occurs at the depth of about 0.75 m, corresponding to the age of ~10.5 ka. Similarly, the MS recorded in Jingchuan shows a large rise from ~9.0 ka to ~6 ka, with calculated rates of 20×10^-8^m^3^/kg per 1 kyr between ~9.0 ka and ~7.5 ka. Then the rates slowed down gradually since ~7.5 ka until the peak of MS value reached. Although a major increase in MS also occurs at Jingchuan, the most rapid rate of change is dated to ~8.5 ka according to the rate-of-change analysis, distinctly later than at Yaoxian. At the northernmost site, Huanxian, the MS values are significantly lower than at Yaoxian and Jianchuan. Here, the increase in MS occurs gradually from ~12 ka to 4 ka, consistent with a minor color change of the loess evident in the field, and it is more difficult to locate objectively the position of the rapid change in MS values than in other sections. Using the rate-of-change analysis, we located the position at ~1.1 m, at an age of ~7.5 ka, broadly in line with the mid-point of the increases in MS values which others have used to determine the boundary [18]. In addition, previous work has suggested that the OSL sensitivity can be used to locate stratigraphic boundaries [18]. The OSL sensitivity in the upper part of the three sections is significantly higher than in the lower parts, which is in close agreement with the boundary obtained from the MS stratigraphy. In summary, there is a clear northwards younging trend in the onset of rapidly changing MS values: ~10.5 ka at the Yaoxian section; ~8.5 ka at the Jingchuan section; and ~7.5 ka at the Huanxian section, in the northwestern part of the CLP.

## Discussion

### The time-transgressive nature of the Pleistocene/Holocene boundary

**Fig 4:**
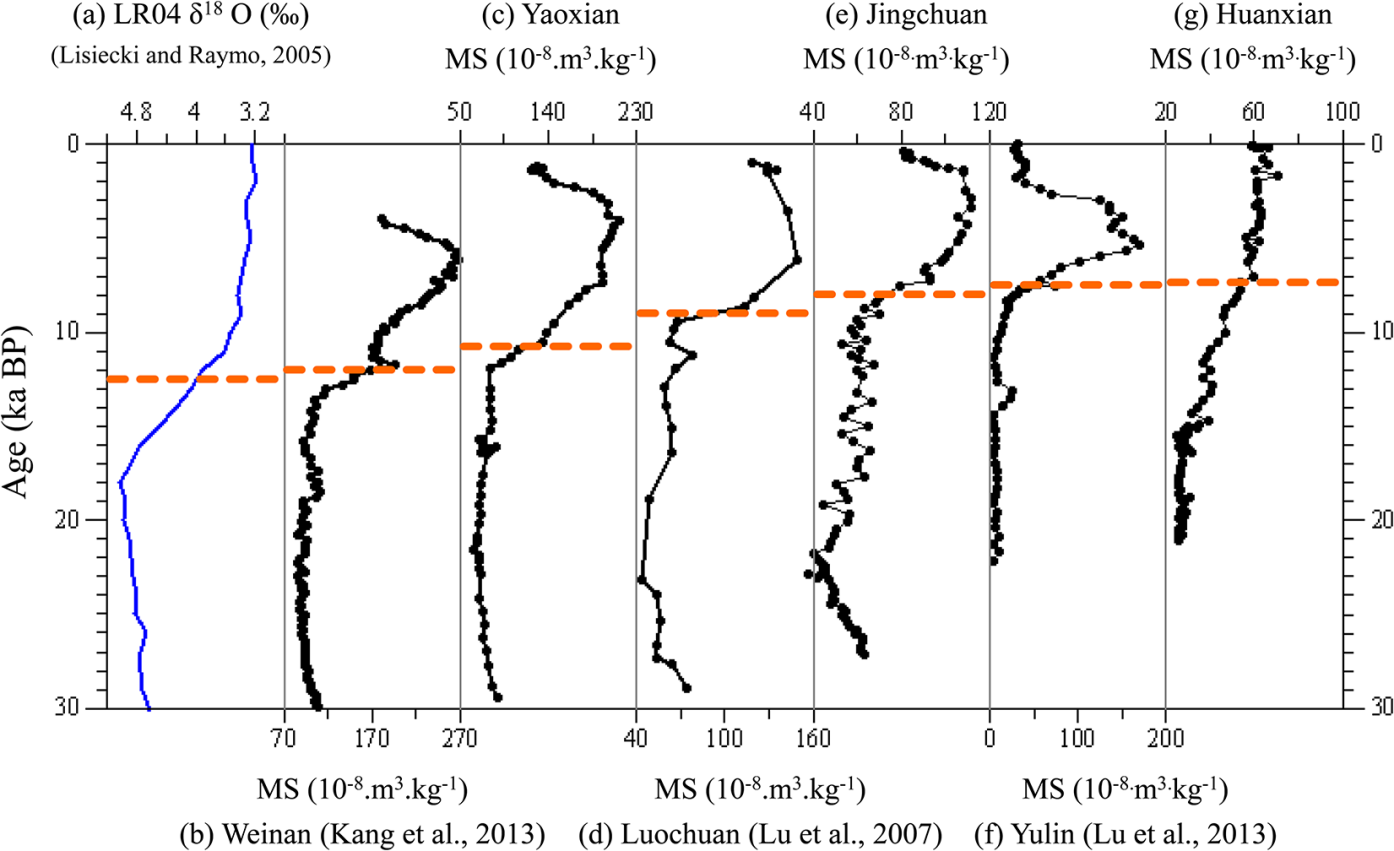
The rapid change in magnetic susceptibility (MS) across the Pleistocene/Holocene transition along a transect from northwest to southeast across the CLP (b-g), and correlation with the LR04 δ^18^O record [27]. b Weinan section [35]; c Yaoxian section; d Luochuan section [17]; e Jingchuan section; f Yulin section [40]; g Huanxian section.

The stratigraphic boundary defined by the major change in MS across the Pleistocene/Holocene boundary has long been assumed to be synchronous in different sections across the CLP, and thus many proxy-age models use this assumption in establishing time series of climate proxies for Chinese loess [14]. To test this assumption, we have located the P/H transition by MS, and dated it using an absolute OSL-based chronology. Comparison of the age of rapid changes in MS in the Yaoxian, Jingchuan and Huanxian sections (Fig 4), indicates that there is a ~3–4 ka lag in the age of onset of large increase in MS across the CLP from the southeast to the northwest.

The earliest marked increase in MS recorded in the studied loess sequences occurred at ~10.5 ka at Yaoxian in the southeast part of the CLP. The timing of the Pleistocene/Holocene boundary at other sections in the southeast of the CLP, based on absolute dating, is broadly in accord with the result for the Yaoxian site. Using rate-of-change analysis, the boundary determined by applying high stratigraphic resolution OSL dating is ~12.0 ka in the Weinan section [35]. The rapid increase in MS using AMS radiocarbon and thermoluminescence dating was determined to be ~12 ka in another Weinan loess record [52]. Similarly, the boundary based on OSL dating of sections at Wulipu and Laoguantai in the southern CLP is dated at ~11 ka [53,54]. These data indicate that the Pleistocene/Holocene transition is dated from ~12 to 10 ka in the southern part of the CLP. In comparison, the age of the boundary at Jingchuan, in the central CLP, ~8.5 ka, is clearly delayed, and this finding is broadly consistent with OSL dates from other sections in this area. Based on OSL dating, the MS values from the Luochuan section exhibit a similar increase as Jingchuan, from ~9.5 ka to ~8.0 ka [17,40]. The L1/S0 transition at the Caihezhan site can be dated to ~8.5 ka, as inferred from OSL ages, equivalent to the timing at the Jingchuan site [55]. Closely spaced optical dating at the Xifeng section also constrains the transition to between ~9.5 ka and 8.0 ka [21,24]. These dates from the central sites yield a mean transition age of 9.0~8.5 ka.

The latest date, ~7.5ka, occurs at the Huanxian section, in the northwestern CLP. This is roughly coincident with OSL dates from the Beiguoyuan section [23], and from the Huanxian section obtained in a previous study [24], as well as at the Yulin section [40]. Although an absolute age error is inevitable, the clear difference of ~3–4 ka between the northwest and southeast demonstrates that the age of the boundary differs significantly across the CLP.

**Fig 5:**
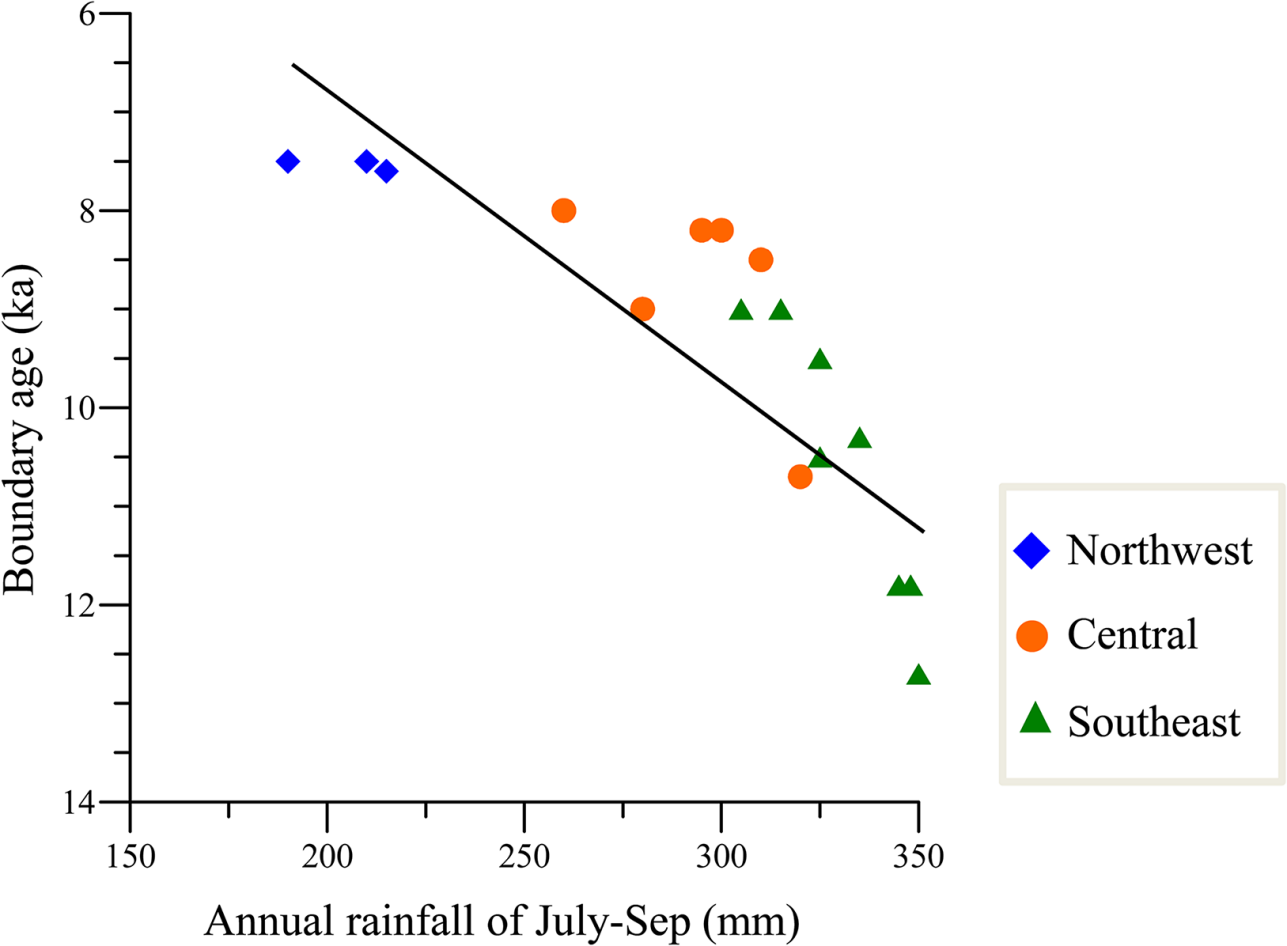
Scatter plot of boundary age as defined by MS stratigraphy versus annual rainfall of July-Sep for different sites. The different symbols used represent the different site locations on the CLP.

In addition, we plotted the boundary age versus location for each of the aforementioned loess sections and the results are shown in Fig 5. We used the annual rainfall of July-Sep as a quantitative expression of the location, since the spatial variation of MAP exhibits a clear gradient across the CLP. There is a significant correlation (R = 0.701) between the timing of the Pleistocene/Holocene boundary and the annual rainfall of July-Sep. Then we performed a simple linear regression analysis, which is the most commonly considered analysis method to examine the statistical relationship between one predictor variable and the response variable. Linear regression analysis reveals spatial gradients with significant slopes (P<0.05) across the CLP. A significant P-value (P<0.05) demonstrates that the boundary age is affected by changes in the annual rainfall of July-Sep. From a statistical point of view, we can conclude that the P/H transition, as recorded in the MS, occurred at different times at different locations across the CLP. Therefore, the determination based on absolute dating, combined with the statistical analysis, confirms that the timing of the transition as defined by the MS exhibits a remarkably strong spatial gradient along a transect from the southeast to the northwest, with the age decreasing from ~12 ka to 8 ka. Therefore, the stratigraphic boundary defined by MS records is time-transgressive across the CLP.

Our study based on OSL dating confirms that the Pleistocene/Holocene boundary as determined by the MS stratigraphy of the CLP is not time-equivalent with the MIS2/1 boundary, with age differences of ~1–4 ka between the MIS boundary (12.05 ka) and the loess boundary (~12.0–7.5 ka). This result is consistent with the studies of the northern and western CLP by Lai and Wintle (2006) [18] and Lu et al. (2006) [24]. Although the Pleistocene/Holocene boundary is defined in a consistent manner, i.e. by the rapid change in a given climatic proxy, the loess boundary defined by the MS is also not well correlated with other records, such as the speleothem δ^18^O record (11.4 ka) [56], and the Greenland ice core record from the North GRIP site (11.7 ka) [57]. Accordingly, the time transgressive MS-defined boundary indicates that the assumption of previous studies that the rapid change in MS values at the L1/S0 stratigraphic boundary of different loess sections can be correlated with the MIS 2/1 boundary, or with the boundary determined by other records, cannot be sustained. Thus the asynchronous boundary defined by the MS is not *sensu stricto* a chronostratigraphic boundary, and therefore alternative methods have been used to define the boundary, such as by using the change in rate of loess accumulation [18,35,58]. On a finer scale, it is difficult to interpret millennial climatic events based on an MS-based age model since this age model overestimates the age of the lowermost sediment by some 1–4 ka; however, it may still be useful for longer time intervals such as an earth orbital time scale. Our study further illustrates the limitation of proxy records such as magnetic susceptibility accurately to derive the chronology of loess deposits, and emphasizes the importance of absolute dating for interrogation these detailed archives of regional climatic and environmental change.

### Possible explanation for the asynchronous MS records

The CLP is located in the East Asian monsoon region. In winter, the cold and dry northwesterly airflows generated by the Siberian High prevail and bring strong winds and cold air to the CLP. In summer, the East Asian summer monsoon carries warm, moist air masses to the CLP, causing heavy rainfall in the area and strong soil development [41]. The desert-loess transitional area is considered to be the northern margin of the present EASM. A progressive and regressive migration history of the East Asian summer monsoon during the Quaternary interglacial-glacial cycles has been previously documented by a number of studies [11,15]. The MS of the loess has long been used as a proxy for pedogenic processes related to the influence of the summer monsoon regime [1,59]. It has been generally accepted that during the Quaternary higher MS values occur during interglacials, and lower values during glacials [59,60], and this finding is confirmed in the present study. Although the processes and factors affecting variations in the MS record of loess-paleosol sequences remain controversial [42,61,62], recent studies have suggested that annual rainfall rather than temperature exerts the dominant effect on soil magnetic enhancement [63,64]. Accordingly, the MS has been used to reconstruct paleoprecipitation in loess sequences [65,66]. Therefore, it is reasonable to suggest that the gradual northward advance of the front of the East Asian summer monsoon rainfall belt is the most plausible driver of the time-transgressive nature of the MS record in the CLP since the last deglacial.

The East Asian summer monsoon (EASM), which is driven by orbitally-induced changes in insolation, carries warm, moist air from the southern oceans to the CLP. The summer monsoon reaches the southeastern margin of the CLP first, and is also much more vigorous in this part of the CLP compared to the northwestern margin. The northern front of the summer monsoon first reached the southern margin of the CLP during at ~12ka, causing heavy rainfall and thus strong soil development. The remarkable change observed in the MS stratigraphy at this time is confirmed by other proxies directly linked to EASM precipitation in sediment archives in the southeastern CLP. A paleoprecipitation reconstruction based on a phytolith record from the Weinan section exhibits a strong increase around 12 ka in the southeastern CLP [10]. Similarly, pedogenic processes inferred from multi-proxies intensified at ~12 ka in the Xingyang section, resulting in the formation of the S0 paleosol [67,68], which is consistent with the rapid increase in MS in the southern CLP.

In contrast, the Huanxian and Jingchuan loess sections, located in the northwestern region of the CLP, were only weakly influenced by the summer monsoon during the early stage of the Pleistocene/Holocene transition. Although solar insolation was enhanced in the northern hemisphere at this time, the ice-sheets in high latitudes of the northern hemisphere remained extensive. The extensive ice-sheets generated strong cold, high pressure air masses and intensified the Siberian High [69,70]. Hence, the East Asian winter monsoon was still strong due to the large gradients in temperature and atmospheric pressure between the polar region and mid-low latitudes. This would impede the northward movement of humid air masses in northern China [71–74], resulting in the humid air masses restricted to the southeastern region of the CLP. This led to a delayed and weaker paleosol development in the north compared to the south. In this case, the MS, used as the proxy of the summer monsoon intensity, may directly document the asynchroneity of the monsoon signal. The northern front of the monsoonal rainfall belt did not penetrate into the northwestern CLP until ~8 ka. The timing of the onset of intense summer monsoon rainfall is supported by estimates of paleorainfall inferred from other loess sections and by lake sediment records from northern China. Paleoecological records from Hulun, Dali and Daihai Lake indicate that precipitation did not increase until ~8 ka in the northern marginal zone of the EASM [75–79]. Similarly, monsoon precipitation based on multi-proxies records significantly increased after ~8 ka in the Yulin loess section [40], which is also located in the northern marginal zone of the EASM. This is also evidenced by the dunes in northern China which maintained a stable phase after ~7–8 ka [80–82]. All of the evidence supports the contention that the intensity of the summer monsoon initially increased in the southern CLP after ~12 ka, but did not reach its maximum intensity, and the monsoonal rainfall belt did not reach its northern limit, until ~8 ka. The distance between the northernmost and southernmost sites is some 300 km, and this can be regarded approximately as the migration distance of the EASM summer monsoon rainfall belt. Clearly, the advance of the air masses may not have been linear.

## Conclusions

In this study, 33 optically stimulated luminescence (OSL) ages were determined in order to date the Pleistocene/Holocene (~MIS 2/1) boundary, as defined by magnetic susceptibility (MS) stratigraphy, at three Chinese loess sections along a southeast-northwest transect across the Chinese Loess Plateau (CLP). The OSL ages demonstrate that a rapid change in MS occurred at ~10.5 ka at Yaoxian in the southeastern CLP; at ~8.5 ka at Jingchuan in the central CLP; and at ~7.5 ka at Huanxian in the northwestern CLP. Thus there is an age discrepancy of ~3–4 ka in the timing of this event between the three sites. These comparisons clearly reveal the time-transgressive nature of changes in the East Asian summer monsoon intensity across the Pleistocene/Holocene transition in the CLP. Our results challenge the previously-held assumption that the boundary age based on MS stratigraphy is time-equivalent in Chinese loess deposits. The time-transgressive nature of the boundary can primarily be explained by the varying intensity of the East Asian summer monsoon across the CLP, which resulted in asynchronous paleosol development at different sites since the last deglacial.
